# Quality assessment of consumer‐facing websites on sodium reduction

**DOI:** 10.1111/jch.14572

**Published:** 2022-09-29

**Authors:** Tasfia Hussain, Taha Ahmedna, Matti Marklund, Lawrence J. Appel, Megan E. Henry

**Affiliations:** ^1^ Krieger School of Arts and Sciences Johns Hopkins University Baltimore Maryland USA; ^2^ Department of Epidemiology Johns Hopkins Bloomberg School of Public Health Baltimore Maryland USA; ^3^ Welch Center for Prevention, Epidemiology and Clinical Research Johns Hopkins University Baltimore Maryland USA; ^4^ The George Institute for Global Health University of New South Wales Sydney Australia; ^5^ Department of Public Health and Caring Sciences Uppsala University Uppsala Sweden; ^6^ Division of General Internal Medicine Johns Hopkins University School of Medicine Baltimore Maryland USA

**Keywords:** diet, Google trends, health information seeking behavior, hypertension, JHU‐SALT, lifestyle modification, nutrition, patient information, patient resources, sodium reduction

## Abstract

The purpose of this paper is to evaluate the quality of information and guidance on dietary sodium reduction available on consumer‐facing websites. Google Trends was used to identify the five most‐used search terms related to dietary sodium reduction. For each term, websites on the first two pages were collected (*n* = 18–20). Of 93 websites collected, 24 were excluded due to defective links, duplicate websites, or not being consumer‐focused. The remaining 69 websites were evaluated using a novel instrument, JHU‐SALT, that includes 14 questions on topics related to salt reduction. The questions are grouped into three domains (“information,” “guidance,” and “accuracy”). For each question, websites were scored using a 3‐step ordinal scale (“topic not addressed,” “topic somewhat addressed,” or “topic addressed adequately”). Only three of 14 JHU‐SALT questions were addressed adequately by a majority of websites. Many websites provided information on the adverse health effects of a high sodium diet (74%, *n* = 51) or mentioned intake recommendations (64%, *n* = 44). Information on fundamental concepts was largely missing. The majority of websites (80%, *n* = 55) provided information on lifestyle strategies to reduce blood pressure, but most did not provide guidance to help implement those strategies. While missing information was common, misinformation was uncommon. The DISCERN questionnaire was utilized as well. Consumers seeking information and guidance on dietary sodium reduction will find that most available websites provide accurate but limited information, and insufficient guidance on how to lower sodium intake. Websites that provide both relevant information and guidance are needed to help consumers effectively reduce dietary sodium.

## 1 INTRODUCTION

An estimated 116 million adults, or more than 45% of the US adult population, were diagnosed with hypertension in 2018.^1^, ^2^ High sodium consumption has been found to raise blood pressure (BP) and is a major risk factor for developing hypertension and BP‐related diseases,^3^, ^4^, ^5^ yet an estimated 89% of adults and 92%–94% of children exceed the *2015–2020 Dietary Guidelines for Americans* for recommended sodium intake of less than 2300 mg of sodium per day.^6^, ^7^, ^8^ Environmental and genetic factors contribute to chronic diseases like hypertension, but changes in diet and lifestyle are considered factors over which individuals have some agency and control.^9^, ^10^, ^11^ Limiting sodium intake is generally recommended by health practitioners^12^ and by public health entities, including the FDA and CDC.^13^

Health information is easily and frequently accessed online, and search engines are utilized by consumers more often than medical societies or libraries.^14^, ^15^, ^16^ In the U.S., 41–42% of households research health information online, while only 23%–25% of US households use the internet to communicate directly with health professionals.^17^ This reliance on online information warrants assessment of the quality of information provided. Evaluations of consumer health information are common, but this has not yet been done for websites designed to assist consumers seeking information on dietary sodium reduction. To understand the quality of easily accessible online sources targeting consumers interested in reducing their sodium intake, we conducted a systematic quality assessment of websites providing information and guidance on sodium reduction.

The main objective of the assessment was to evaluate the quality of information and guidance on dietary sodium reduction available on consumer‐facing websites.

## 2 METHODS

### 2.1 Search strategy

A list of 14 potential search terms was compiled. These included terms that consumers might enter in an internet search engine if they wished to lower their sodium intake (Appendix I). The five most popular search terms in the US for the period between 04/05/2020 and 04/06/2021 were determined from this list by utilizing the Google Trends feature of the Google search engine. Google trends present search terms not as absolute hits, but as an “average interest over time” relative to other search terms. Interest is defined by Google as the proportion of all searches on all topics at a certain time and region and is indexed to a value of 100, with 100 indicating peak popularity.^18^ To assess the relative interest among websites, we standardized their interest as a percentage of the peak popularity among the terms.

Once the five primary search terms were identified, each was entered into a Google search. To limit location and previous browsing history biases, location services were turned off; all browser cache, cookies, and history were cleared; all Web and application history was deleted; and incognito mode was used.

### 2.2 Inclusion and exclusion criteria

Websites listed on the first two pages of each Google search were collected (*n* = 18–20 websites per search term) based on evidence that most consumers do not go past the first two pages on a search.^19^ Defective links and duplicate websites were excluded. Peer‐reviewed scientific review papers were also excluded as these are not designed for (nor always accessible to) consumers.

### 2.3 Website evaluation

Each website was evaluated using two instruments. An instrument was needed to assess the availability and quality of information and guidance that a website offers to consumers seeking to lower their sodium intake, but no such instrument existed. A team including physicians, dietitians, nutritionists, and other public health researchers at the Johns Hopkins Bloomberg School of Public Health with expertise in sodium reduction developed an instrument with 14 questions, termed the **JHU‐Sodium Assessment Learning Tool** (**JHU‐SALT**) (Appendix II). Each question was categorized under the broader domains of “information,” “guidance,” or “accuracy”. For each question, websites were scored using a 3‐step ordinal scale from 0 to 2 (“none,” “somewhat,” or “adequate” information provided). A composite score for each website was created through the sum of scores for each question. The maximum possible composite score was 28 (14 questions with scores from 0 to 2).

The second instrument was the DISCERN questionnaire which is a generic instrument used to assess the quality of consumer information regarding healthcare treatment choices.^20^ DISCERN includes 15 questions assessing general consumer health information with a focus on treatment. None of the DISCERN questions are specifically related to sodium reduction. For each question in DISCERN, the website was scored using a scale of 1 (no), 2 (partially no), 3 (partially), 4 (partially yes), and 5 (yes). The questionnaire has an additional question for overall website score. This final question was rated 1, 3, or 5, as per instructions.

Assessments using JHU‐SALT and DISCERN were made independently by two researchers (TH and TA). The two researchers adjudicated differences, and any unresolved disagreements were adjudicated by a third investigator (MEH).

## 3 RESULTS

### 3.1 Popular search terms

**FIGURE 1:**
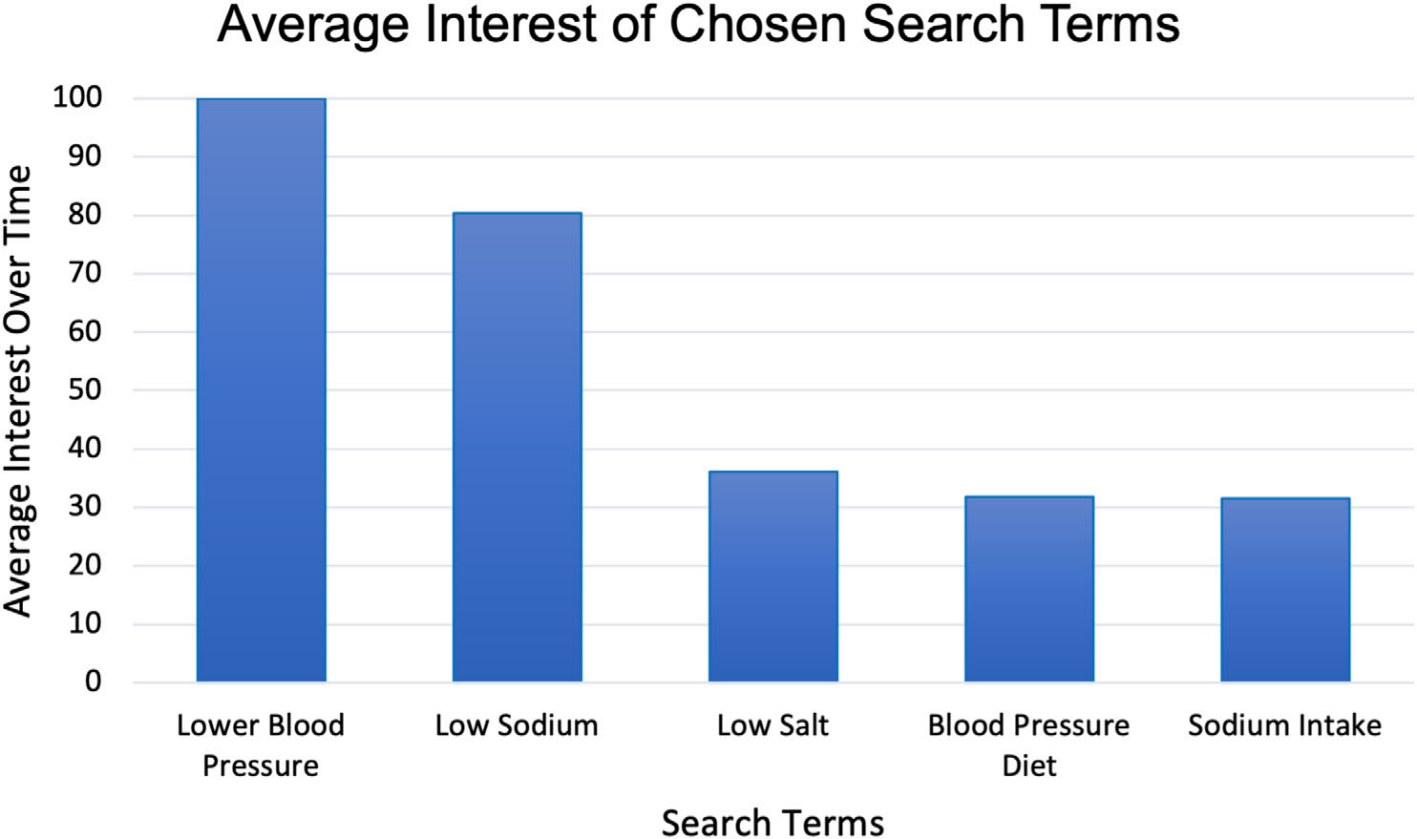
Average interest of five most popular search terms. Average interest is a metric used by Google. Interest is defined by Google as the proportion of all searches on all topics at a certain time and region and is indexed to a value of 100, with 100 indicating peak popularity. 'Lower blood pressure' is the reference category with a value of 100.

**FIGURE 2:**
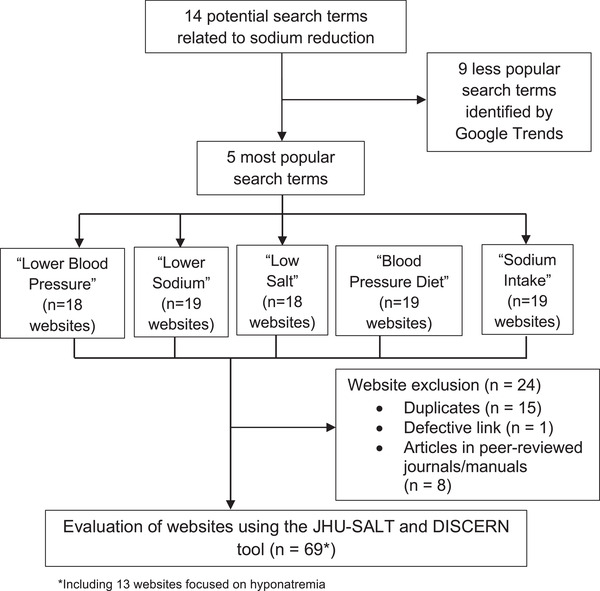
Search strategy used to identify final *n* = 69 websites for evaluation. Google Trends was utilized to identify the five most popular search terms among 14 potential terms. Websites from the top two Google pages for each of the most popular search terms were pooled.

The five most popular terms (in descending order) were: “Lower Blood Pressure,” “Low Sodium,” “Low Salt,” “Sodium Intake,” and “Blood Pressure Diet” (Figure 1). Among these search terms, “Lower Blood Pressure” was set as the reference in Google Trends (100% popularity). The next most popular was “Low Sodium” (71% popularity). The remaining three terms were considerably less popular (each ≤18%). Of the 93 websites identified from the five search terms, one defective link, 15 duplicate websites, and eight peer‐reviewed websites were excluded, leaving 69 websites that were included in this evaluation (Figure 2).

### 3.2 JHU‐SALT results

**FIGURE 3:**
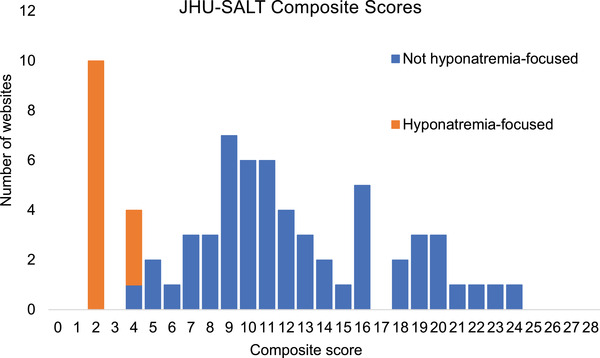
Composite scores of *n* = 69 websites evaluated by JHU‐SALT. The scores are stratified based on whether or not the website was hyponatremia‐focused. A composite score is defined as the sum of the scores for each question on JHU‐SALT with a maximum possible composite score of 28.

**FIGURE 4:**
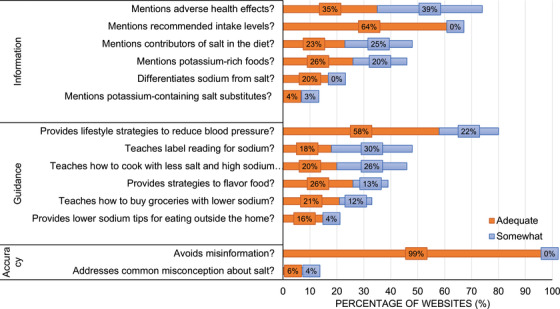
Percentage of adequate or somewhat adequate scoring websites for each JHU‐SALT question. Each bar excludes the percentage of websites that received a score of not adequate for that question. Percentages were calculated out of a total of *n* = 69 websites.

From the total sample of included websites (*n* = 69), the median and range of overall scores for JHU‐SALT was 10 and 2–24, respectively. The highest score was 24 (*n* = 1). Only seven websites had a score of 20 or more while one in three websites scored ≤ 8 out of 24 (Figure 3). Of all 14 JHU‐SALT questions, only four were addressed “somewhat” or “adequately” by more than half of the websites, the four being “mentions adverse health effects,” “mentions recommended intake levels,” “provides lifestyle strategies to reduce blood pressure,” and “avoids misinformation.” (Figure 4). Few websites provided adequate guidance (≥8 out of 12, *n* = 14) or adequate information (≥8 out of 12, *n* = 8), and only three websites scored well in all three domains of JHU‐SALT (ie, “information,” “guidance,” and “accuracy”). Of the 14 websites with adequate guidance, only three websites had adequate information. Of the eight websites with adequate information, only three websites had adequate guidance.

An unexpected finding from the extraction of websites was the large number of hyponatremia‐related websites identified in the searches (*n* = 13, 19% of all included websites). These websites were returned as search results from using the search terms “Low Salt” and “Low Sodium”. These websites scored appropriately poorly in the JHU‐SALT evaluation (scores ≤ 4 out of 28), as they were not focused on providing information or guidance on dietary sodium reduction.

### 3.3 Evaluation of information

More than half of websites mentioned the adverse health effects of high sodium diets (*n* = 51, 74%) and recommended maximum sodium intake levels (*n* = 44, 64%). However, a minority of websites provided information on major dietary sources of salt (*n* = 33, 47%) or potassium‐rich foods (*n* = 32, 46%); explained the relationship between the terms ‘sodium’ and ‘salt’ (*n* = 14, 20%); or mentioned potassium‐containing salt substitutes (*n* = 5, 7%) (Figure 4).

### 3.4 Evaluation of guidance

More than half of websites provided lifestyle strategies to reduce blood pressure (80%, *n* = 55). Fewer than half of websites provided guidance on how to read labels (48%, *n* = 33), taught how to cook with less salt and high sodium ingredients (46%, *n* = 32), or provided strategies to flavor food with ingredients other than salt (39%, *n* = 27). Only a small number of websites provided guidance on how to buy groceries with low sodium content (33%, *n* = 23) or select low sodium options while eating out (20%, *n* = 14) (Figure 4).

### 3.5 Evaluation of accuracy

Very few websites addressed common misconceptions about salt (10%, *n* = 7), but almost all websites avoided misinformation (99%, *n* = 68) (Figure 4).

### 3.6 DISCERN results

After applying the DISCERN questionnaire to the health problem of high sodium, the results indicated that most of the selected websites contained a high quality of information on sodium reduction treatments which was generally reliable, detailed, and trustworthy. Of the 15 DISCERN questions, 12 questions were addressed adequately or somewhat adequately by a majority of the websites (Appendix III).

## 4 DISCUSSION

In this systematic quality assessment of consumer‐facing websites on sodium reduction, the most popular websites provided either information or guidance on dietary sodium reduction, but rarely both. A small number of websites scored well on all domains of JHU‐SALT (information, guidance, and accuracy) and can be recommended to individuals seeking information online to lower their sodium intake (Table 1).

**TABLE 1:** Top three websites evaluated by JHU‐SALT

	Scores					
Rank	Website link	Organization	Information (out of 12)	Guidance (out of 12)	Accuracy (out of 4)	Composite (out of 28)
1	https://www.fda.gov/food/nutrition‐education‐resources‐materials/sodium‐your‐diet	FDA	8	12	4	24
2	https://www.mayoclinic.org/healthy‐lifestyle/nutrition‐and‐healthy‐eating/in‐depth/sodium/art‐20045479	Mayo Clinic	10	10	3	23
3	https://www.uptodate.com/contents/low‐sodium‐diet‐beyond‐the‐basics	Up to Date	8	12	2	22

The composite score is calculated by the sum of the information, guidance, and accuracy scores.

To our knowledge, no prior study has been conducted to assess the quality of consumer‐facing information for sodium reduction online. Previous environmental scans have assessed the quality of information disseminated by patient‐education websites with health conditions such as chronic kidney disease and migraines.^21^, ^22^, ^23^ To our knowledge, this is the first quality assessment of consumer‐oriented sodium reduction websites and the only one that specifically evaluated both information and guidance.

Our study used a general evaluation instrument (DISCERN) and a targeted instrument (JHU‐SALT) to assess the quality of sodium reduction resources available to consumers. DISCERN was useful in demonstrating the reliability of public health guidance provided by most sources, confirming that most websites were broadly useful and trustworthy. However, DISCERN was developed to evaluate information on medical treatments. Given the general nature of the questions in DISCERN and the topic of sodium reduction, which is not a medical therapy but rather a strategy based on lifestyle change, DISCERN was less relevant than JHU‐SALT.

JHU‐SALT was designed to evaluate the information and guidance available on consumer‐facing websites in regard to sodium reduction in the diet. It was able to identify both the relative quantity and quality available on various websites. Only a small portion of evaluated websites excelled in providing key information and guidance on reducing dietary sodium intake. Though nearly all sites contained accurate information, no site adequately addressed all topics JHU‐SALT identifies as important for learning about and achieving dietary salt reduction. Even among the highest‐scoring websites, information was consistently found to be inadequate even though guidance and accuracy were excellent. Among low‐scoring websites, while accuracy was excellent, both information and guidance were consistently found to be inadequate. Our findings are consistent with a previous study investigating the quality of online information on pulmonary arterial hypertension; the quality of information available to consumers was found to be generally inadequate, with poor transparency and reliability.^24^

Additionally, there was a lack of overlap between top‐scoring websites in guidance and top‐scoring websites in information. This reveals an opportunity for improvement in the available resources for consumers to access comprehensive information on the same site.

A surprising finding was that among all 69 included websites, nearly 20% were focused on hyponatremia, a condition with an estimated prevalence in the US of 1.7%.^25^ In comparison, nearly 50% of all adult Americans have hypertension, and the prevalence is likely higher among consumers interested in (or instructed to) reduce their sodium intake. Consumers could find the conflicting information between sodium reduction and the risks of hyponatremia confusing or even discouraging.

Our study has limitations. First, the Google search engine was used to input search terms. Although caches were cleared and incognito mode was used, results could potentially differ upon replication due to the use of other browsers or unknown or unmodifiable elements of the Google search engine. Second, the popularity of search terms used to collect websites is subject to change, highlighting the need for repeated monitoring and evaluation. Third, differences in interpretation did arise between the investigators, although a standardized procedure for adjudication and reconciliation produced an agreement.

Strengths of this study include the development and utilization of an instrument targeted toward the assessment of sodium reduction websites. JHU‐SALT should be further assessed for reliability and validity, but it was shown to be useful in ranking website quality in terms of information, guidance, and accuracy; and for identifying both the most useful and irrelevant resources available online. It can be used to guide website content development to better support individuals seeking comprehensive support for dietary sodium reduction, as well as follow‐up assessments of website content. The methodology of website collection and evaluation followed in this study is also highly adaptable to other regions, languages, health conditions, and lifestyle interventions.

Our study has implications for website creators, as well as health care providers and the general public. Website creators should provide consumers with information regarding the health consequences of high sodium intake and the benefits of sodium reduction, along with practical and concrete guidance and tips on how sodium reduction can be achieved. The components of JHU‐SALT could be utilized as a checklist to ensure the completeness of information and guidance. Websites focusing on hyponatremia should clarify that the information provided regarding hyponatremia is targeted to certain patient groups and that for most individuals (outside these specific groups) reducing sodium intake is associated with significant health benefits. Healthcare providers may utilize the top three scoring websites in this evaluation to recommend to patients for sodium reduction information, as they represent the most complete and accurate information available currently (Table 1).

In conclusion, consumers seeking information and guidance online will find that the most easily accessible websites offer accurate but limited information and provide insufficient guidance on how to lower sodium intake. Websites that provide practical tools and promote relevant skills are needed to help consumers effectively reduce dietary sodium.

## AUTHOR CONTRIBUTIONS

All authors conceived and designed the study. Tasfia Hussain and Taha Ahmedna collected the data, analyzed the data, and wrote the initial draft of the manuscript. Matti Marklund, Megan E. Henry, and Lawrence J. Appel offered insights into analysis of data and interpretation. All authors contributed to the manuscript revision and approved the final version of the manuscript.

## CONFLICT OF INTEREST

Lawrence J. Appel, MD receives payments from Wolters Kluwer for chapters in UpToDate on the relation of blood pressure with weight, exercise, smoking, and sodium intake.

## APPENDIX I

List of potential search terms checked through Google Trends (most to least popular)

1. Lower blood pressure (reference, 100%)
2. Low sodium (71%)
3. Low salt (17%)
4. Sodium intake (12%)
5. Blood pressure diet (9%)
6. Blood pressure sodium (7%)
7. Blood pressure salt (6%)
8. Salt intake (4%)
9. Reduce salt (2%)
10. Reduce sodium (2%)
11. How to lower sodium (2%)
12. How to lower salt (1%)
13. Lower sodium intake (< 1%)
14. Lower salt intake (< 1%)

## APPENDIX II

### JHU‐SALT

This instrument was created to assess websites on guidance and information in regard to sodium reduction in diet.

Questions 1–6 assess information while questions 7–12 assess guidance. Questions 13 and 14 assess accuracy.

Score the following questions with either 0(No), 1(Somewhat), or 2(Adequate).

Questions:

1. Does the website mention adverse health effects of sodium consumption?
2. Does the website mention recommended sodium intake levels?
3. Does the website mention contributors of salt in the diet?
4. Does the website mention potassium‐rich foods?
5. Does the website differentiate sodium from salt?
6. Does the website mention potassium‐containing salt substitutes?
7. Does the website provide lifestyle strategies to reduce blood pressure?
8. Does the website teach label reading for sodium?
9. Does the website teach how to cook with less salt and high sodium ingredients?
10. Does the website provide strategies to flavor food without salt?
11. Does the website teach how to buy groceries with lower sodium?
12. Does the website provide lower sodium tips for eating outside the home?
13. Does the website avoid misinformation?
14. Does the website address common misconceptions about salt?

## APPENDIX III

### DISCERN questionnaire evaluation

Bar graph with adequate or somewhat adequate responses to each question on the DISCERN tool.
